# Representation of Sound Objects within Early-Stage Auditory Areas: A Repetition Effect Study Using 7T fMRI

**DOI:** 10.1371/journal.pone.0124072

**Published:** 2015-05-04

**Authors:** Sandra Da Costa, Nathalie M.-P. Bourquin, Jean-François Knebel, Melissa Saenz, Wietske van der Zwaag, Stephanie Clarke

**Affiliations:** 1 Service de Neuropsychologie et de Neuroréhabilitation, Département des Neurosciences Cliniques, Centre Hospitalier Universitaire Vaudois, Université de Lausanne, Lausanne, Switzerland; 2 National Center of Competence in Research, SYNAPSY—The Synaptic Bases of Mental Diseases, Service de Neuropsychologie et de Neuroréhabilitation, Département des Neurosciences Cliniques, Centre Hospitalier Universitaire Vaudois, Université de Lausanne, Lausanne, Switzerland; 3 Laboratoire de Recherche en Neuroimagerie, Département des Neurosciences Cliniques, Centre Hospitalier Universitaire Vaudois, Université de Lausanne, Lausanne, Switzerland; 4 Centre d’Imagerie BioMédicale, Ecole Polytechnique Fédérale de Lausanne, Lausanne, Switzerland; Harvard Medical School/Massachusetts General Hospital, UNITED STATES

## Abstract

Environmental sounds are highly complex stimuli whose recognition depends on the interaction of top-down and bottom-up processes in the brain. Their semantic representations were shown to yield repetition suppression effects, i. e. a decrease in activity during exposure to a sound that is perceived as belonging to the same source as a preceding sound. Making use of the high spatial resolution of 7T fMRI we have investigated the representations of sound objects within early-stage auditory areas on the supratemporal plane. The primary auditory cortex was identified by means of tonotopic mapping and the non-primary areas by comparison with previous histological studies. Repeated presentations of different exemplars of the same sound source, as compared to the presentation of different sound sources, yielded significant repetition suppression effects within a subset of early-stage areas. This effect was found within the right hemisphere in primary areas A1 and R as well as two non-primary areas on the antero-medial part of the planum temporale, and within the left hemisphere in A1 and a non-primary area on the medial part of Heschl’s gyrus. Thus, several, but not all early-stage auditory areas encode the meaning of environmental sounds.

## Introduction

The human primary auditory cortex (PAC) is currently defined on the basis of cytoarchitectonic and tonotopic criteria. It is co-extensive with the koniocortex on the medial two-thirds of Heschl’s gyrus (HG) [1–6]. Its precise localization in activation studies relies, especially in cases of partial or complete HG duplication (in 40–60% of hemispheres [4,7]), on tonotopic mapping. The presence of orderly tonotopic representations is a key feature of the three core areas in non-human primates [8–16], where primary subfields are organised in anterior-posterior frequency gradients from high-to-low (caudal primary auditory subfield A1), low-to-high (rostral primary auditory subfield R), and high-to-low (rostrotemporal primary auditory subfield RT) frequencies, with a low frequency cluster at the boundary between A1 and R and a high frequency cluster between R and RT. In humans, fMRI studies consistently revealed a double frequency representation with anterior-posterior frequency gradients on HG which are of the human homologues of monkey A1 and R subfields [17–23], but less often the third reversal equivalent to the RT subfield.

The cortex adjacent to PAC contains several non-primary areas—the auditory belt areas—which are architectonically inhomogeneous [12,15,16,24,25]. The use of function-related chemical stains on post-mortem human brains has led to the identification of several early-stage areas which are the likely homologues of primate belt areas: the anterior area (AA), the medial area (MA), the anterior-lateral area (ALA), the lateral area (LA) and the posterior area (PA) [5,6]. The non-primary areas are partly frequency-selective, but without a clearcut tonotopic organisation [17]. They tend to respond to more complex auditory stimuli, which characterise them as putative belt areas [26].

Recognition of environmental sounds involves the early-stage auditory areas as well as parts of the temporal and parietal convexities [27–31]. The discrimination between broad categories, such as living vs. man-made sound sources occurs very rapidly (as early as 70 ms after stimulus onset) due to the interactions of top-down and bottom-up processes, which characterize the ventral and dorsal auditory streams [32–35]. Several lines of evidence indicate that early-stage areas on the supratemporal plane (STP) analyze spectrotemporal features of sounds, whereas higher-order areas on the temporal convexity are dedicated to semantic processing [36,37], suggesting a hierarchy within the ventral stream. Thus, our ability to distinguish between different samples of the same sound object (broad category) relies on the fundamental differences in sound amplitude and spectral power. We recognize the mewing of a kitten from that of an old cat because of these differences even though we already categorized the sound as coming from a cat. Additional evidence points to a key role of the planum temporale (PT) in the analysis of complex auditory stimuli, including auditory scene analysis [32,38].

In the visual system, the hierarchical organisation of the ventral and dorsal streams has been investigated extensively with repetition suppression (or adaptation) paradigms, where stimulus repetition induces a reduction of neural activity. The repetition suppression effects are related to a stimulus-induced adaptation of the underlying neuronal population for a preferred feature, which diminishes when a new, unpreferred stimulus is presented [39–44]. Repetition effects occur during a narrow time window, ~100–200 ms post stimulus onset, as demonstrated in electro- and magnetoencephalography (EEG: [34,45–49]; MEG: [50]). Stimulus-induced adaptations of neural populations have been also captured by fMRI as changes of the BOLD signal during repeated presentations (fMRI: [36,44,51–55]). Several of the previously mentioned studies suggested that repetition priming effects due to the semantic content of sounds, and not merely to their acoustic features, occur within STP [20,26,28,36,37,47,48,51,52,54–66]. Because of technical constraints, these studies did not analyze PAC, belt and parabelt areas separately: data were acquired at low [54,56,65] or high field strengths [20,36,37,45,51,55,57,59,63,67,68], with rather large voxel sizes (2–3 mm or more) and spatial smoothing (2 to 8 mm FHWM).

Here, we have made use of the high spatial resolution of ultra-high field fMRI to investigate the representations of environmental sounds within individual early-stage auditory areas by means of a repetition effect paradigm. The increased signal-to-noise ratio and BOLD signal, the decrease of the signal strength of venous blood (due to the short relaxation times) and the restriction of activation signals to the cortical gray matter were shown to improve spatial specificity [69,70]. All together, these technical advances are beneficial for the tonotopic mapping of the (small) human areas A1 and R and for repetition effect paradigms within individual early-stage areas which require high sensitivity [17,18,20,71–74].

We addressed two issues. First, as suggested by previous low-spatial resolution repetition effect studies, the planum polare (PP), which belongs to the hierarchically organized ventral pathway, may encode the meaning, and not solely the acoustic features of environmental sounds. It is currently not clear whether this is already the case in PP belt areas, i. e., areas immediately adjacent to PAC, and possibly also in PAC. If so, this would challenge the key tenet of a strictly hierarchical model of sound recognition. Second, PT, which serves as hub for complex auditory processing, may encode the meaning of environmental sounds. The possible role of PT belt and/or parabelt areas has not yet been investigated.

## Materials and Methods

### Subjects

Ten subjects (6 female, mean age 23.9 ± 3.7) with normal hearing and no history of neurological or psychiatric illness participated in the study. Written, informed consent forms were signed by all subjects after a brief oral description of the protocol. The Ethics Committee of the Faculty of Biology and Medicine of the University of Lausanne approved all experimental procedures. Eight subjects were right-handed, one left-handed and one ambidextrous. One subject’s data were discarded due to large motion artefacts. Data from the remaining nine subjects were used in the current analysis.

### Auditory stimuli

Sound stimuli were generated using MATLAB and the Psychophysics Toolbox (www.psychtoolbox.org). Stimuli were delivered binaurally via MRI-compatible headphones (Insert Earphones, SensiMetrics, MA, USA) featuring flat frequency transmission from 100 Hz to 8 kHz. Sound intensities were adjusted to match standard equal-loudness curves (ISO 226) at phon 95: the sound intensity of each pure tone stimulus (ranging from 88 to 8000 Hz) was adjusted to approximately equal perceived loudness of a 1000 Hz reference tone at 95 dB SPL (range of sound intensities: 87–101 dB SPL). Sound levels were further attenuated (~35 dB) by silicone ear plugs (Etymotic Research Inc., ER38-15SM). All subjects were debriefed after the session and all reported hearing sounds clearly over the background of scanner noise.

### Tonotopic mapping

Pure tones (88, 125, 177, 250, 354, 500, 707, 1000, 1414, 2000, 2828, 4000, 5657, and 8000 Hz; half-octave steps with a sampling rate of 44.1 kHz) were presented in ordered progressions, following our previously described protocols [17,75]. Each subject performed two tonotopic runs with ascending and descending progressions (low to high and high to low frequencies, respectively). Pure tone bursts were presented during a 2 s block in consecutive steps until all 14 frequencies had been presented. The 28 s progression was followed by a 4 s silent pause, and this 32 s cycle was repeated 15 times per 8 min run. Resulting maps of the two runs were averaged. This paradigm is designed to induce travelling waves of response across cortical tonotopic maps [76]. Linear cross-correlation was used to determine the time-to-peak of the fMRI response wave on a per-voxel basis, and to thus assign a corresponding best frequency value to each voxel. Analyses were performed in individual-subject volumetric space and results were then projected onto same-subject cortical surface meshes.

**Fig 1:**
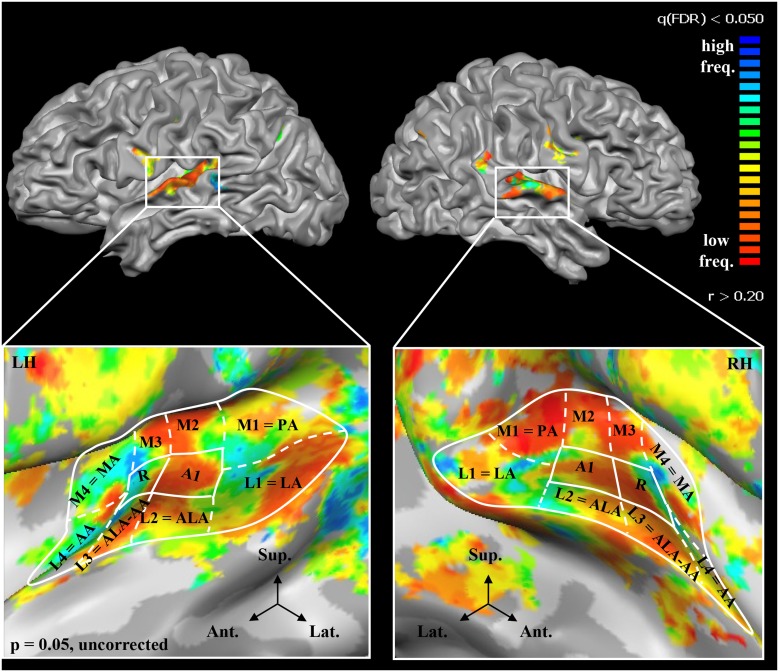
Tonotopic maps in the left and right hemisphere. (LH, RH) of a typical subject in the lateral view of the hemispheres (FDR corrected: 0.05, r>0.20; top) and enlarged, in upper view of the unfolded STP (bottom). In each hemisphere, two mirror-symmetric gradients, corresponding to the primary areas A1 and R, are located on HG. The surrounding, frequency-selective region (p = 0.05, uncorrected) was subdivided into 8 ROIs: *M1*, *L1*, *M2*, *L2*, *M3*, *L3*, *M4*, and *L4*. Several ROIs were homologues of the auditory areas found in previous architectonic studies [5,6]. M1, L1, L2, L3, L4 and M4 corresponded, respectively, to PA (posterior auditory area), LA (lateral auditory area), ALA (anterior lateral auditory area), AA (anterior auditory area), ALA—AA (junction between ALA and AA), and MA (medial auditory area).

Similar to the example shown in Fig 1, two tonotopic gradients with mirror symmetry ("high-low-low-high") were clearly observed in both hemispheres of all subjects [17–21,75,77–79], A1 was defined by the more posterior "high-to-low" gradient and R by the more anterior "low-to-high" gradient. In macaque auditory cortex, fields A1 and R receive parallel thalamic input and are both considered part of the primary auditory core.

### fMRI repetition suppression experiment

The selection of the stimuli used proceeded as follows. To start with, we chose 583 extracts of easily identifiable sound objects, often heard in everyday life, from BBC sound effects database (following [46,47]) using Adobe Audition (Adobe Systems Software Ireland Ltd.). The duration of each sound was 500 ms. Amplitudes, sampling frequencies and linear rise/fall times were normalized with the same routine for all sounds (16 bits, 44.1 kHz, 50 ms rise/fall times, without high-pass/low-pass filtering). Monophonic sounds were duplicated into stereophonic sounds. Five normal subjects, which did not participate in the fMRI study, were asked whether the sound was clear, whether they recognized it, and then to name it and to rate the degree of confidence of their recognition. We then proceeded to select sounds which were correctly named by all five subjects and which reached high confidence level (4 or 5 on a scale of 0–5). Sounds were then sorted into two groups: the repetition group (REP group, i.e. eight different individual/exemplars of the same sound) and the control group (CTRL group, i.e. eight different sounds objects). The two groups were compared for familiarity and for acoustic properties (amplitude and spectral power). The degree of familiarity, i.e. the level of confidence with which the subjects judged their recognition, was equivalent in both groups. The acoustics characteristics were controlled with the same approach as described previously [80]. Randomly selected sounds from either group were compared for their acoustic characteristics (amplitude and spectral power) at each time point with unpaired t-tests. This iteration was repeated until two lists of sounds were identified with less than 1% of significantly different time points (p<0.05); ~ 1000 iterations were performed). To limit false negatives, i.e. to avoid underestimating putative differences, we did not apply any correction for multiple comparisons. To minimize the differences between REP and CTRL sets, we set the threshold to 1% the time points. This procedure yielded a total of 323 environmental sounds (64 REP sounds and 259 CTRL sounds). As an additional control measure, we calculated the mean power spectrum (amplitude spectrum) of each condition (see S4 Fig) and performed an unpaired t-test on those, which revealed no significant differences between the two conditions. Although the overall sound acoustics were controlled between REP and CTRL groups, within each REP and CTRL blocks, the amplitude and the spectral power differed between sounds repeats (see S4 Fig). Semantic categories (animal vocalizations, human-made sounds, tools, music instruments, and natural scene-like sounds; see S1 Table for the list of sounds used in the experiment) were equally distributed in both groups. Sounds from the REP group were never repeated in the CTRL group, and sounds from the CTRL group were randomised between blocks, subjects and runs.

Subjects listened passively to sounds during fMRI acquisitions, with their eyes closed. A block design with alternating blocks of sounds of the same semantic category (REP) and sounds of different semantic categories (CTRL) was used. REP blocks were made of eight different repetitions of the same object (e.g. eight baby cries of different babies), with in total 8 REP blocks or 64 REP sounds per run. CTRL blocks had 8 different exemplars of different categories randomly selected at the beginning of each run (8 different objects x 8 blocks = 64 out of the 259 CTRL sounds). Sounds were presented bilaterally during 500 ms with an inter-stimulus interval of 1500 ms during 16 s, followed by a 14 s silent pause. Each fMRI run consisted of 16 blocks of 30 s (8 REP and 8 CTRL, 8 minutes in total). Two runs, with the same sequence of sounds, were acquired both before and after tonotopic mapping runs. Sound presentations were synchronized with the scanner trigger. All subjects reported clear perception and recognition of the stimuli of both groups.

### MRI data acquisition and analysis

Imaging was performed with an actively shielded 7 Tesla Siemens MAGNETOM scanner (Siemens Medical Solutions, Erlangen, Germany) located at the Centre d’Imagerie BioMédicale (CIBM) in Lausanne, Switzerland. Functional data were acquired using a 32-channel head volume rf-coil (Nova Medical, USA [81]) and an EPI pulse sequence with sinusoidal read-out (1.5 x 1.5 mm in-plane resolution, slice thickness = 1.5 mm, TR = 2000 ms, TE = 25 ms, flip angle = 47°, slice gap = 1.57 mm, matrix size = 148 x 148, field of view 222 x 222, 30 oblique slices covering the superior temporal plane, first three EPI volumes discarded). The sinusoidal shape of the readout gradients reduces the acoustic noise produced by the scanner. A T1-weighted high-resolution 3-D anatomical image was acquired for each subject using the MP2RAGE pulse sequence optimized for 7T (resolution = 1 x 1 x 1 mm, TR = 5500 ms, TE = 2.84 ms, TI1 = 2350 ms, TI2 = 0 ms, slice gap = 1 mm, matrix size = 256 x 240, field of view = 256 x 240; [82]).

Preprocessing steps were performed with BrainVoyager QX v2.3 software and included standard linear trend removal, temporal high-pass filtering and motion correction, but no spatial smoothing. Functional time-courses were interpolated into a 1 x 1 x 1 mm volumetric space and registered to each subject’s 3D Talairach-normalized anatomical dataset. Cortical surface meshes were generated from each subject’s anatomical scan using automated segmentation tools of the program. Alignment of anatomical data across subjects was performed with the cortex-based alignment [83]. This is a non-rigid alignment of cortical surface meshes across individuals based on the gyral and sulcal folding patterns. Each subject’s cortical surface meshes were aligned to a target mesh (separately for left and right hemispheres) which had an intermediate HG anatomy (partial HG duplication in the left hemisphere and a large single gyrus in the right hemisphere). All alignments were visually inspected. A group-averaged contrast *environmental sound vs*. *rest activation map* was generated in this cortex-based aligned mean space during the data analysis of the repetition suppression experiment (for regions outside the auditory cortex, see S3 Fig).

### Identification of non-primary early-stage auditory areas

Individual tonotopic mappings were used to identify in each subject non-primary early stage areas, defined as subject-specific regions of interest (ROIs). This approach has been previously used in several studies across different modalities (visual localizer: [84]; auditory localizer: [36,85]). Maps were set to an intermediate threshold (r>0.13, equivalent to p≤0.05, uncorrected) in order to cover a region including most of STP in all subjects and hemispheres. We then manually outlined a contiguous patch of interest (mean volume LH: 1400.87 mm^2^ ± 321.35, and mean volume RH: 1364.58 mm^2^ ± 189.15) of cortical surface including the two tonotopic gradients within PAC, the remaining medial and lateral parts of HG, the posterior part of the PP and the anterior part of the PT using the drawing tools in BrainVoyager QX (external outlines in Fig 1). This patch of interest was subdivided into 10 regions with the following steps. First, the primary areas A1 and R were localized based on mirror-symmetric preferred-frequency reversals. The anterior and posterior borders thereof were drawn along the outer high-frequency representations, while lateral and medial borders were set so as to cover only the medial two-thirds of HG (in accordance with human architectonics [5,86]). The border between A1 and R was then drawn along the long axis of the low-frequency cluster. The location of the border of A1 and R was not dependent on the correlation threshold. Second, we divided the non-primary region surrounding A1 and R into eight ROIs. The common border between A1 and R was extended until the outlines of the main patch, dividing the main patch into anterior and posterior parts. The same was done for the anterior and posterior borders of the primary subfields. This resulted in six subfields on HG (A1, R, M2, M3, L2, and L3), two on the anterior part of PT (M1/L1) and two on the posterior part of PP M4/L4). The 10 areas (M1, L1, M2, A1, L2, M3, R, L3, M4, and L4; Fig 1, Table 1 and S2 Fig) included thus primary and non-primary auditory areas, in agreement with the monkey model [8]. Several of these areas have been identified in previous architectonic studies [5,6] (Table 1); M1, L1, L2, L3, L4 and M4 corresponded, respectively, to PA (posterior auditory area), LA (lateral auditory area), ALA (anterior lateral auditory area), ALA—AA (junction between the anterior lateral and anterior auditory areas), AA (anterior auditory area), and MA (medial auditory area).

**Table 1:** Mean Talaraich coordinates of all ROIs.

Labels	Talairach coordinates [X Y Z] ± std			Mean area (mm^3^) ± std			Area
*Right hemisphere*							
right A1	[44 ± 4	-20 ± 3	9 ± 2]	145.71	±	27.00	PAC
right R	[44 ± 4	-21 ± 3	10 ± 2]	133.35	±	31.58	PAC
right L1	[56 ± 4	- 28 ± 4	12 ± 3]	212.07	±	126.06	LA
right L2	[57 ± 3	- 17 ± 3	7 ± 3]	125.97	±	24.99	ALA
right L3	[52 ± 4	-10 ± 3	4 ± 2]	148.14	±	20.16	ALA—AA
right L4	[44 ± 4	-10 ± 5	0 ± 3]	213.67	±	97.77	AA
right M1	[46 ± 4	-30 ± 2	16 ± 3]	136.31	±	33.29	PA
right M2	[38 ± 3	-29 ± 2	16 ± 3]	67.35	±	20.57	-
right M3	[34 ± 2	-25 ± 2	14 ± 3]	65.75	±	20.41	-
right M4	[35 ± 3	-20 ± 2	7 ± 4]	119.92	±	71.61	~ MA
*Left hemisphere*							
left A1	[-40 ± 3	-24 ± 3	9 ± 2]	138.67	±	30.27	PAC
left R	[-39 ± 3	-21 ± 3	9 ± 2]	106.00	±	25.05	PAC
left L1	[-52 ± 4	-29 ± 4	11 ± 3]	246.53	±	69.66	LA
left L2	[-52 ± 3	-18 ± 3	6 ± 2]	140.07	±	38.11	ALA
left L3	[-47± 4	-11 ± 4	4 ± 2]	165.28	±	38.47	ALA—AA
left L4	[-40 ± 4	-12 ± 6	0 ± 3]	209.44	±	105.22	AA
left M1	[-40 ± 3	-34 ± 3	14 ± 3]	155.71	±	59.12	PA
left M2	[-34 ± 2	-30 ± 2	15 ± 3]	62.58	±	22.41	-
left M3	[-31 ± 2	-26 ± 2	15 ± 3]	59.66	±	15.78	-
left M4	[-32 ± 2	-20 ± 2	8 ± 4]	100.23	±	48.17	~ MA

Mean Talaraich coordinates of all ROIs with standard deviations and their corresponding areas defined by cytoarchitechtonic studies [5,6]. Area: corresponding area; PAC: primary auditory cortex; LA: lateral auditory area; ALA: anterior lateral auditory area; AA: anterior auditory area; PA: posterior area; MA: medial auditory area.

These regions of cortical surfaces were projected into the same-subject’s 1 x 1 x 1 mm interpolated volumetric space to generate 3D ROIs with a width of 2 mm (-1 mm to 1 mm from the vertex centre). Individual time-courses from the 3D-ROIs were subsequently analyzed in the repetition effect experiment.

### Time-course analysis and plateau definition

Functional individual time-courses were also extracted for all individual voxels within the main region of interest. Using home-made Matlab scripts, they were baseline corrected and averaged in space (within ROIs) and in time (across runs and blocks repetitions), separating conditions, in order to have two final time-courses, one for REP and one for CTRL, with 15 time points each per ROI, for each hemisphere and subject. These time-courses were then averaged across subjects and normalised to the first time-point.

Repetition suppression effects measured with EEG are related to amplitude differences between the first presentation and the repeat of a brief, single event, which can be picked up due to the high temporal resolution of the technique. Repetition-induced changes of neural activity are more difficult to assess with fMRI, due to its poor temporal resolution. In order to overcome this limitation in our study, we used a block design approach. We assumed that whether the sound was followed by a repetition or not, the hemodynamic response of the first sound had the same behaviour at onset, and only the plateau (or the saturation period) differed between CTRL and REP conditions. We hypothesized that in case of repetition effects, i.e. in REP blocks, the slope of the BOLD response will be steeper than during the CTRL condition. BOLD signal intensities of consecutive time frames were subtracted pair wise to calculate their relative slopes (t_n + 1_–t_n_). We tested our hypothesis on the slope values using paired t-tests against 0. Positive p values indicate a rise period, negative values a decay and null values a plateau. We restricted our results in time to a minimum of two consecutive time frames. Time frame by time frame paired t-tests revealed significant differences (p<0.05, uncorrected) in slopes during the same time periods for all conditions and hemispheres: a rise between 2–6 s, a plateau between 6–18 s, and a decay between 18–22 s (S1 Fig).

## Results

### Identification of early-stage auditory-related areas

Individual phase-encoding analysis of the time-courses of the tonotopy runs reproduced the mirror-symmetric tonotopic gradients as previously reported in other studies [17,75]. The location and the extent of frequency-selective regions were determined individually in each hemisphere and subject. When calculated at the same statistical threshold (p<0.05, uncorrected), this region covered in each subject a large part of the STP, including PP and PT, and it was co-extensive with the region activated by environmental sounds (main effect of environmental sound presentation; see S2 Fig). Here tonotopic mapping was used as localizer for primary and non-primary auditory areas for which repetition effects to environmental sounds were then investigated.

The primary areas A1 and R were identified by their characteristic “high-low-low-high” reversal. The surrounding frequency-selective region was parcelled into 8 ROIs and designated L1-L4 on the lateral and M1-M4 on the medial part (Fig 1; see Materials and Methods). Several ROIs corresponded to areas identified in previous architectonic studies ([5,6]; L1 = LA, L2 = ALA, L3 = ALA-AA, L4 = AA, M1 = PA, M4 = MA).

### Repetition effects within auditory cortex on the supratemporal plane

**Fig 2:**
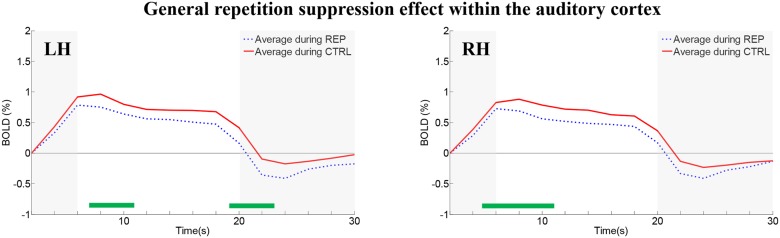
Mean of the group average time-courses of the REP and CTRL conditions within the auditory cortex on the STP (defined by main effect of environmental sound) in the left and right hemisphere (LH, RH). Significant differences between conditions are highlighted by the green bars in the bottom of the graphs (paired t-test, p<0.05, uncorrected). Gray shading denotes the rise and decay periods (for definition see Supporting Information).

Irrespective of the condition (REP, CTRL), the BOLD time course within the auditory cortex had the similar evolution, consisting of rise, plateau and decay. These three time windows were defined by means of temporal derivatives of the average time course (Fig 2 and S1 Fig). The rise comprised the period of 2–6 s after block onset and was very likely shaped by the hemodynamic response to the first sound. The plateau stretched over the period of 6–18 s and was shaped by the hemodynamic response to the 7 following stimuli. A significant difference between REP and CTRL during the plateau was interpreted as a repetition suppression effect and hence an indication that the neural population encoded the meaning of the stimuli.

In a first analysis the BOLD response was averaged over the whole STP region with significant main effect of environmental sounds (which was co-extensive with the 10 early-stage areas). The auditory cortex time-courses (averaged across blocks and subjects for each condition) were significantly different between conditions near the peak of the BOLD response (which also correspond to the beginning of the plateau period) in both hemispheres (p<0.05, uncorrected; green line in Fig 2). Bilateral REP time-courses peaked 2 s earlier (6 and 8 s after stimulus onset for REP and CTRL times-courses, respectively) and had a different plateau decrease, than the CTRL time-courses. This could possibly reflect different saturation of the BOLD response when different individuals/exemplars of the environmental sounds are presented. Left hemisphere CTRL time-courses showed a sustained plateau between 12 and 18 s after stimulus onset, whereas right CTRL time-courses showed a slow decay. REP time-courses showed a faster return to baseline or lower plateau than CTRL time-courses, comparable to previous results with other sensory modalities showing repetition suppression effects [40]. The REP and CTRL conditions differed significantly at 8–10 s in the left hemisphere and at 6–8 s in the right hemisphere (paired t-test, uncorrected). No significant difference was found between the hemispheres for either condition.

### Repetition effects within individual early-stage auditory areas

**Fig 3:**
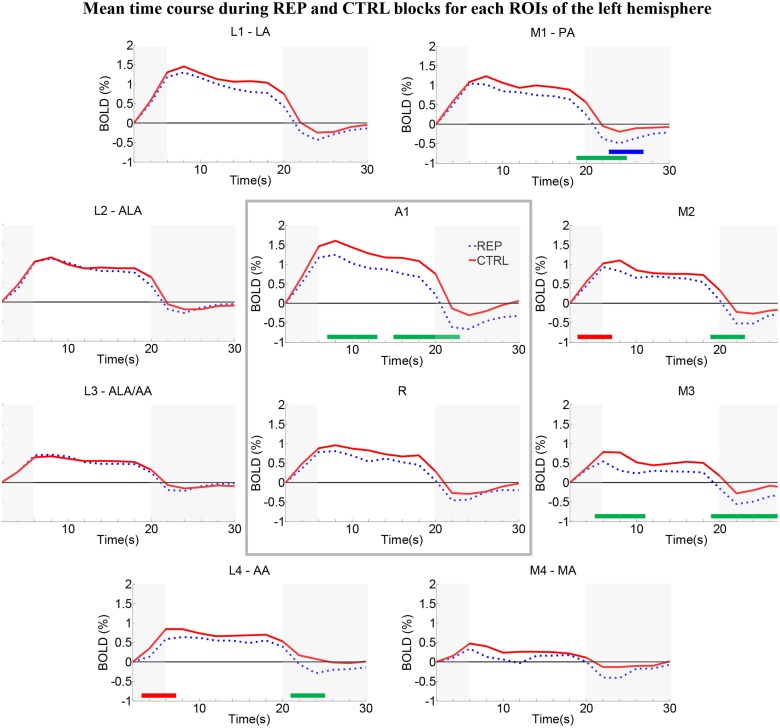
Group average time-courses of the two conditions in the left hemisphere within individual early-stage areas. BOLD signal changes (in %) were plotted across time points of the block. REP blocks tended to yield lower activation than CTRL blocks. Significant differences between conditions and hemispheres are highlighted by green (CTRL > REP), red (CTRL LH > CTRL RH) and blue bars (REP RH > REP LH) at the bottom of each graph (paired t test, p<0.05, uncorrected).

**Fig 4:**
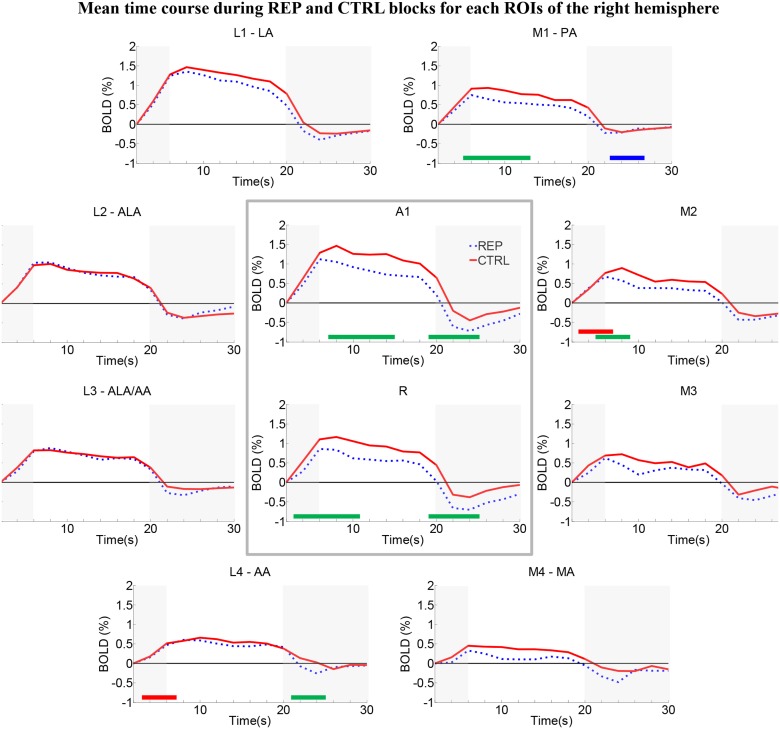
Group average time-courses of the two conditions in the right hemisphere within the auditory cortex ROIs. BOLD signal changes (in %) were plotted across time points of the block. REP blocks tended to yield lower activation than CTRL blocks. Same conventions as in Fig 3.

Time-courses of the BOLD response were analyzed separately in each area (Figs 3 and 4). Independent of the condition, responses look larger in posterior than anterior areas (for CTRL in RH: L1 > A1 > R > L2 > M1 > M2 > L3 > M3 > L4 > M4; for CTRL in LH: A1 > L1 > M1 > L2 > M2 > R > L4 > M3 > L3 > M4; for REP in RH: L1 > A1 > L2 > L3 > R > M1 > M2 > M3 > L4 > M4; for REP in LH: L1 > A1 > L2 > M1 > M2 > R > L3 > L4 > M3 > M4; see S3 Table). The REP and CTRL time-courses were almost identical in areas L2 and L3 on either hemisphere (paired t-test, p<0.05, uncorrected; see S3 Table), suggesting that these areas are insensitive to semantic repetition effects. In several areas the REP time-courses peaked earlier than CTRL (RH: L1; LH: M1: both: A1, R, M2 and M3; paired t-test, p<0.05, uncorrected; see S3 Table). This could possibly reflect faster saturation of the BOLD response in these regions when different individuals/exemplars of the environmental sounds are presented.

The repetition effect, i.e., a significant difference between the REP and CTRL conditions during the plateau phase, was present in areas A1 and M3 of the left hemisphere (Fig 3) and in A1, R, M1 and M2 of the right hemisphere (Fig 4).

**Fig 5:**
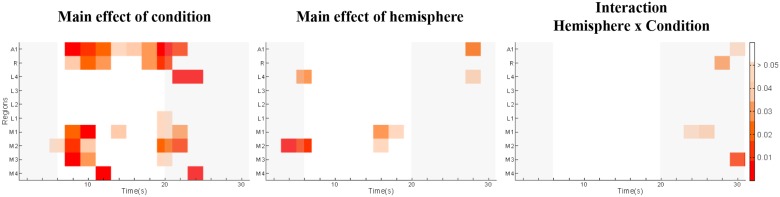
Time frame by time frame 2-way ANOVA condition (REP, CTRL) x hemisphere (RH, LH) for each of the 10 early stage areas (A1, R, L1, L2, L3, L4, M1, M2, M3, M4). Gray shading highlights the rise and decay periods, red hues denote significant effects at a given time point and area. During the plateau phase a significant main effect of condition was present in A1, R, M1, M2, M3 and M4 as well as a significant main effect of hemisphere at isolated time points in M1 and M2.

A time-point-per-time-point 2 x 2 ANOVA (Hemisphere x Condition) on the BOLD time-courses revealed a main effect of condition during the plateau phase in 6 areas, A1, R, M1, M2, M3, M4 and L4 (Fig 5, left panel, p<0.05, uncorrected). A main effect of hemisphere was present during the plateau phase in 2 areas, M1 and M2 (Fig 5, middle panel, p<0.05, uncorrected). No significant interaction Hemisphere x Condition was observed during the plateau phase (Fig 5, right panel).

### Environmental sounds representations on the posterior temporal convexity

Main effect of environmental sounds, irrespective of condition, revealed bilateral activation clusters in the superior temporal gyrus (STG), near the HG, and two clusters in the right middle temporal gyrus (ES3 and ES4, S2 Fig and S2 Table). BOLD responses tended to be larger in ES3 than ES4. However, neither of these regions showed a significant difference between REP and CTRL conditions.

## Discussion

**Fig 6:**
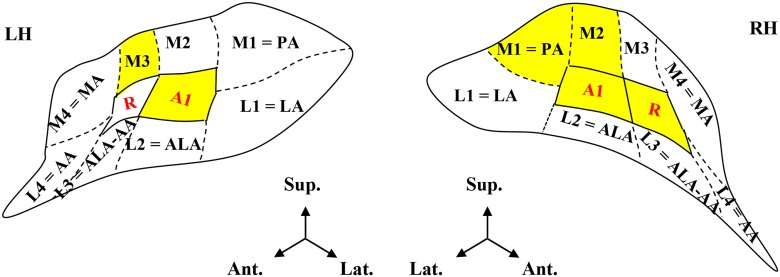
Early-stage auditory areas with repetition suppression effect, i. e. significant difference between the REP and CTRL conditions during the plateau phase (gray shading).

Our results indicate that the representations of the meaning of environmental sounds are already present at the level of early-stage auditory areas. The repeated presentation of eight acoustically different sounds of the same semantic entity yielded repetition suppression effects in areas A1, R, M1 and M2 in the right hemisphere and in A1 and M3 in the left hemisphere (Fig 6). No repetition effects were observed in the other 6 areas on the right and 8 areas on the left side. Interestingly, the putative belt areas on the PP, often associated with the ventral auditory pathway, do not appear to encode the meaning of environmental sounds, whereas the primary cortex and the belt areas on the medial part of the PT do so.

### Semantic coding in the ventral auditory stream occurs outside PP belt areas

Our results showed that the meaning of environmental sounds is encoded at the level of early-stage auditory areas within the PT, but not within the belt areas on the PP. This supports a model of hierarchical processing within the antero-ventral recognition pathway. Seminal studies have shown that the ventral auditory stream is dedicated to the identification of sound sources and that it processes sound-to-meaning in a hierarchically organized fashion. Regions on the STP, close to PAC, were found to be selective for acoustic features of stimuli such as spectral structure and temporal variability, but not for stimulus category, whereas more anterior regions on STG presented category selective responses [37]. The role of regions outside STP in category-specific coding has been reported also in other studies using comparisons between sounds of living vs man-made sources [34], animals vs tools [87] or several different categories [59,63,88,89]. The semantic involvement of the temporal convexity, but not STP, has been further demonstrated by means of repetition effects for specific sound categories (vocalizations: [50–52,90–92] or specific sound objects [46,47,49]). One seminal study reported category specific adaptation effects on the STP, but not specifically within belt areas [55]; neural responses to animal and tool sounds were acquired with 3T fMRI (spatial resolution of 3mm, spatial smoothing of 8 mm) and adaptation effects were averaged in anatomically pre-defined regions HG, PP, PT, anterior STG and posterior STG in the right and left hemisphere. The PP as delimited in this analysis stretched up to the temporal pole, reaching far beyond the belt areas. PP, HG and PT yielded adaptation effects to tool sounds on the left side and to tool and animal sounds on the right side.

We would like to stress that the absence of repetition effects in PP belt areas is not due to a lack of sensitivity of our paradigm. Repetition effects were well present in distinct PAC and PT areas (Fig 6) and also when the whole PAC-PP-PT region was averaged (Fig 2).

### The role of the planum temporale in the representation of sound objects

The presence of repetition effects within PAC and within belt areas on the medial PT suggests that the meaning of environmental sounds is encoded at very early stages of cortical processing. The PT as a whole was shown to encode category-specific information of sounds as fine-grained patterns of distributed activity [68,93]. More generally PT was shown to encode both pitch and spatial information [45,94–97] or recognition and spatial information [28,30]; it has been referred to as a hub for the processing of different sound attributes [98]. Several studies have highlighted its role in auditory scene analysis, i.e., in the segregation of concurrent auditory streams by means of pitch or spatial differences [38,99,100]. The separation of meaningful sounds in an acoustically complex environment, as assessed by the task to localize a target sound among four simultaneous distracters vs alone, was shown to involve PT, together with the left inferior frontal gyrus, the precuneus and the right STG [101].

Taken together, the above evidence indicates that the PT plays an important role when sound recognition occurs in a complex acoustic environment. Surprisingly, the meaning of environmental sounds is already represented at the level of belt areas on the PT but not on the PP, which belongs to the classical recognition pathway.

The representation of sound objects on PT may constitute a processing stream for sound recognition which is, at least partially, independent from the antero-ventral pathway. The existence of a dual semantic pathway is supported by three observations. First, functional and anatomical studies speak in favour of an early segregation between the two semantic pathways. Semantic information is already encoded in PAC ([55] and here), which has strong connections to belt areas [102]. The most parsimonious explanation is that PAC shares semantic information with the postero-medial belt areas on the PT semantic and acoustical information with the anterior belt areas on PP. The alternative explanation that the belt areas on PT receive input from the anterior belt areas and constitute thus the next step in semantic processing cannot be excluded, but it is not supported by the connectivity patterns between the human core, belt and parabelt areas [102]. Second, the nature of the semantic representation differs between the two pathways. The PT has been shown to play an important role in combining semantic and spatial information [45,94–97], whereas the antero-ventral pathway mediates a truly position-independent coding [46,64]. Third, a relative functional independence of the two pathways is suggested by patient studies which reported a double dissociation between deficits in auditory scene analysis such as supported by the PT and semantic identification of sound objects in cases of focal brain lesions [103].

### Methodological considerations

The subdivision of the supratemporal plane, which we used in this study, not based on anatomical landmarks, but on identification of PAC by the presence of mirror symmetric frequency reversals [17,75,77]. Recent improvements in T1 mapping at ultra-high field allowed a definition of PAC in each individual hemisphere according the underlying myelin layout of the cortex [73,74]. These seminal studies demonstrate a very good overlap of tonotopic maps and highly myelinated core on HG in selected cases and propose a potentially unique method for the localization of PAC. Before using a combination of these methods systematically as PAC localizer, it will be necessary to assess systematically the combination of tonotopy and myelin contrast in cases of HG variants of partial or complete duplication. Previous studies have shown that PAC, as defined by the dual tonotopic maps, is not restricted by the sulcal pattern of HG [17]. Instead, there is a continuum between the different variants and the tonotopic maps, where PAC extends over both parts of the gyrus in case of duplications.

### Conclusions

Repetition effects revealed the encoding of the meaning of environmental sounds within primary areas A1 and R as well two belt areas on the antero-medial part of the PT in the right hemisphere and within A1 and a belt area on the medial part of HG in the left hemisphere, but not within belt areas on the PP. These results speak in favour of a dual auditory semantic pathway, one within the hierarchically organized antero-ventral stream and the other within the PT. The latter, but not the former, encodes the meaning of environmental sounds already at the level of belt areas.

## Supporting Information

S1 Fig Time frame by time frame t-tests. A—B. Plateau definition. Temporal derivatives of the averaged time-courses for each ROI illustrate the slope between two consecutive time–points. Paired t-tests of the derivatives against 0 pointed out three different periods: rise from 2 to 6 s, plateau from 6 to 18 s, and decay from 18 to 22 s. Shades of blue correspond to positive slopes (rise), orange-red to negative slopes (decay) and white to zero-gradient parts of the curves.
(TIF)

S2 Fig Auditory cortex ROIs with underlying anatomy. For this exemplar subject, the left hemisphere had a single HG and the right hemisphere a complete duplication. 10 ROIs were defined based on the tonotopic gradients for each hemisphere: *M1*, *L1*, *M2*, *A1*, *L2*, *M3*, *R*, *L3*, *M4*, *and L4*. Several ROIs corresponded to the auditory areas found in the architectonic studies of Rivier and Clarke (1997) and Wallace et al. (2002). Blue line: anterior Heschl’s gyrus border; green line: posterior Heschl’s gyrus border; red line: intermediate sulcus.
(TIF)

S3 Fig Main effect of environmental sound presentations. The group average fixed-effect multi-subject GLM contrast *environmental sounds vs*. *rest* (i.e. REP + CTRL > rest) revealed activation outside the auditory cortex (S2 Fig, ES1, ES2) on both sides in the posterior superior temporal gyrus (STG), posterior middle temporal gyrus (MTG; S2 Fig and S1 Table; ES3 area: 362.59 mm^2^ and ES4 area: 308.11 mm^2^; p<0.05, Bonferroni correction). As for individual ROIs, the group ROIs were labelled with their region name and projected into the reference brain 1 x 1 x 1 mm interpolated volumetric space. Individual time courses of these regions were subsequently analyzed in the repetition suppression experiment. Time-courses of ES3 and ES4 are plotted for each condition in the graph. ES3 and ES4 ROIs showed both the same tendency, with higher BOLD response during control blocks, but none showed significant differences. It is to be noted that the group average fixed-effect multi-subject GLM constrast REP vs CTRL did not show any significant difference (p>0.05, Bonferroni correction). Upper panel: significant activation clusters (p<0.05, Bonferroni corrected). Lower panels: enlargement of the activated regions on a partially inflated brain. Environmental sounds activated two large clusters within the STG (ES1 and ES2), but also two smaller clusters in the right posterior MTG (ES3 and ES4). Mean time courses for these latter clusters are plotted in red and green in the graph between the two enlargements. Time frame by time frame analysis revealed no significant differences between the two conditions surviving the inclusion criteria.
(TIF)

S4 Fig **A. Mean amplitude spectrum of the environmental sounds used in the paradigm.** Each amplitude spectrum of the sounds of the two conditions has been decomposed using a fast Fourier transform function and plotted across the frequency range from 0 to 25000 Hz. Blue line: mean amplitude spectrum for the repetition group sounds; red line: mean amplitude spectrum for the control group sounds. Unpaired t-tests between the amplitude spectra of both conditions for each frequency revealed that 110 non-consecutive frequencies were significantly different between conditions, which corresponded to 1% after Bonferroni correction (110/11025 = 0.01). **B. Amplitude spectrum of each sound in a REP block where eight different bell sounds were presented.** Frequency distributions within a block are different in each exemplar compared to the mean amplitude spectrum of REP condition (bottom right graph).
(TIF)

S1 Table Environmental sounds used in the repetition suppression paradigm. Only sounds correctly recognized during the sound recognition pilot by five subjects were used in the fMRI experiment. All sounds of the REP group (8 sound objects) were used in the fMRI runs, whereas only one exemplar of each sound object was randomly selected in the CTRL group (64 sounds objects). The REP groups was the same in all subjects, whereas the CTRL group varied in all subjects. human voc.: human vocalizations; human non-voc.: human non-vocalizations; env. sound: environmental sound.
(DOCX)

S2 Table Main effect of the environmental sound presentation (REP + CTRL > silence). Centre coordinates of the activation clusters shown in S3 Fig, t scores, and p values. Only regions that remained significant at p<0.05 after application of the Bonferroni correction were considered.
(DOCX)

S3 Table Maxima, minima and amplitudes of the BOLD response during REP and CTRL in both hemispheres. Paired t-tests between REP *vs* CTRL maxima (*[max]*) revealed significant differences in right A1^(1)^, right M1^(2)^, right M2^(3)^, right M4^(4)^, left A1^(5)^, and left M3^(6)^ (p<0.05, uncorrected). Paired t-tests between REP *vs* CTRL minima (*[min]*) revealed significant differences in right R^(7)^, left A1^(8)^ and left M3^(9)^ (p<0.05, uncorrected). Paired t-tests between RH *vs* LH maxima during REP and RH *vs* LH minima during CTRL revealed significant differences in M2^(10)^ and L2^(11)^, respectively (p<0.05, uncorrected). No significant differences were found for the amplitudes.
(DOCX)

## References

[1] Clarke S, Rivier F. Compartments within human primary auditory cortex: evidence from cytochrome oxidase and acetylcholinesterase staining. Eur J Neurosci. 1998;10: 741–745. 9749735 10.1046/j.1460-9568.1998.00043.x

[2] Galaburda A, Sanides F. Cytoarchitectonic organization of the human auditory cortex. J Comp Neurol. 1980;190: 597–610. 10.1002/cne.901900312 6771305

[3] Morosan P, Rademacher J, Schleicher A, Amunts K, Schormann T, Zilles K. Human Primary Auditory Cortex: Cytoarchitectonic Subdivisions and Mapping into a Spatial Reference System. NeuroImage. 2001;13: 684–701. 10.1006/nimg.2000.0715 11305897

[4] Rademacher J, Morosan P, Schormann T, Schleicher A, Werner C, Freund HJ, et al Probabilistic mapping and volume measurement of human primary auditory cortex. NeuroImage. 2001;13: 669–683. 10.1006/nimg.2000.0714 11305896

[5] Rivier F, Clarke S. Cytochrome oxidase, acetylcholinesterase, and NADPH-diaphorase staining in human supratemporal and insular cortex: evidence for multiple auditory areas. NeuroImage. 1997;6: 288–304. 10.1006/nimg.1997.0304 9417972

[6] Wallace MN, Johnston PW, Palmer AR. Histochemical identification of cortical areas in the auditory region of the human brain. Exp Brain Res Exp Hirnforsch Expérimentation Cérébrale. 2002;143: 499–508. 10.1007/s00221-002-1014-z 11914796

[7] Marie D, Jobard G, Crivello F, Perchey G, Petit L, Mellet E, et al Descriptive anatomy of Heschl’s gyri in 430 healthy volunteers, including 198 left-handers. Brain Struct Funct. 2013; 10.1007/s00429-013-0680-x PMC4341020 24310352

[8] Baumann S, Petkov CI, Griffiths TD. A unified framework for the organization of the primate auditory cortex. Front Syst Neurosci. 2013;7: 11 10.3389/fnsys.2013.00011 23641203 PMC3639404

[9] Baumann S, Griffiths TD, Rees A, Hunter D, Sun L, Thiele A. Characterisation of the BOLD response time course at different levels of the auditory pathway in non-human primates. NeuroImage. 2010;50: 1099–1108. 10.1016/j.neuroimage.2009.12.103 20053384 PMC2880247

[10] Brugge JF, Merzenich MM. Responses of neurons in auditory cortex of the macaque monkey to monaural and binaural stimulation. J Neurophysiol. 1973;36: 1138–1158. 4761724 10.1152/jn.1973.36.6.1138

[11] Hackett TA, Preuss TM, Kaas JH. Architectonic identification of the core region in auditory cortex of macaques, chimpanzees, and humans. J Comp Neurol. 2001;441: 197–222. 11745645 10.1002/cne.1407

[12] Kaas JH, Hackett TA. Subdivisions of auditory cortex and processing streams in primates. Proc Natl Acad Sci. 2000;97: 11793–11799. 10.1073/pnas.97.22.11793 11050211 PMC34351

[13] Morel A, Kaas JH. Subdivisions and connections of auditory cortex in owl monkeys. J Comp Neurol. 1992;318: 27–63. 10.1002/cne.903180104 1583155

[14] Morel A, Garraghty PE, Kaas JH. Tonotopic organization, architectonic fields, and connections of auditory cortex in macaque monkeys. J Comp Neurol. 1993;335: 437–459. 10.1002/cne.903350312 7693772

[15] Petkov CI, Kayser C, Augath M, Logothetis NK. Functional Imaging Reveals Numerous Fields in the Monkey Auditory Cortex. PLoS Biol. 2006;4: e215 10.1371/journal.pbio.0040215 16774452 PMC1479693

[16] Rauscheckear JP, Tian B, Pons T, Mishkin M. Serial and parallel processing in rhesus monkey auditory cortex. J Comp Neurol. 1997;382: 89–103. 9136813

[17] Da Costa S, van der Zwaag W, Marques JP, Frackowiak RSJ, Clarke S, Saenz M. Human Primary Auditory Cortex Follows the Shape of Heschl’s Gyrus. J Neurosci. 2011;31: 14067–14075. 10.1523/JNEUROSCI.2000-11.2011 21976491 PMC6623669

[18] Formisano E, Kim DS, Di Salle F, van de Moortele PF, Ugurbil K, Goebel R. Mirror-symmetric tonotopic maps in human primary auditory cortex. Neuron. 2003;40: 859–869. 14622588 10.1016/s0896-6273(03)00669-x

[19] Humphries C, Liebenthal E, Binder JR. Tonotopic organization of human auditory cortex. NeuroImage. 2010;50: 1202–1211. 10.1016/j.neuroimage.2010.01.046 20096790 PMC2830355

[20] Moerel M, De Martino F, Formisano E. Processing of natural sounds in human auditory cortex: tonotopy, spectral tuning, and relation to voice sensitivity. J Neurosci Off J Soc Neurosci. 2012;32: 14205–14216. 10.1523/JNEUROSCI.1388-12.2012 PMC6622378 23055490

[21] Striem-Amit E, Hertz U, Amedi A. Extensive cochleotopic mapping of human auditory cortical fields obtained with phase-encoding FMRI. PloS One. 2011;6: e17832 10.1371/journal.pone.0017832 21448274 PMC3063163

[22] Talavage TM, Sereno MI, Melcher JR, Ledden PJ, Rosen BR, Dale AM. Tonotopic organization in human auditory cortex revealed by progressions of frequency sensitivity. J Neurophysiol. 2004;91: 1282–1296. 10.1152/jn.01125.2002 14614108

[23] Woods DL, Alain C. Functional imaging of human auditory cortex. Curr Opin Otolaryngol Head Neck Surg. 2009;17: 407–411. 10.1097/MOO.0b013e3283303330 19633556

[24] Hackett TA, de la Mothe LA, Camalier CR, Falchier A, Lakatos P, Kajikawa Y, et al Feedforward and feedback projections of caudal belt and parabelt areas of auditory cortex: refining the hierarchical model. Audit Cogn Neurosci. 2014;8: 72 10.3389/fnins.2014.00072 PMC4001064 24795550

[25] Tian B, Reser D, Durham A, Kustov A, Rauschecker JP. Functional specialization in rhesus monkey auditory cortex. Science. 2001;292: 290–293. 10.1126/science.1058911 11303104

[26] Chevillet M, Riesenhuber M, Rauschecker JP. Functional correlates of the anterolateral processing hierarchy in human auditory cortex. J Neurosci Off J Soc Neurosci. 2011;31: 9345–9352. 10.1523/JNEUROSCI.1448-11.2011 21697384 PMC3142575

[27] Lewis JW, Wightman FL, Brefczynski JA, Phinney RE, Binder JR, DeYoe EA. Human Brain Regions Involved in Recognizing Environmental Sounds. Cereb Cortex. 2004;14: 1008–1021. 10.1093/cercor/bhh061 15166097

[28] Viceic D, Fornari E, Thiran J-P, Maeder PP, Meuli R, Adriani M, et al Human auditory belt areas specialized in sound recognition: a functional magnetic resonance imaging study. Neuroreport. 2006;17: 1659–1662. 10.1097/01.wnr.0000239962.75943.dd 17047449

[29] Adriani M, Maeder P, Meuli R, Thiran AB, Frischknecht R, Villemure J-G, et al Sound recognition and localization in man: specialized cortical networks and effects of acute circumscribed lesions. Exp Brain Res Exp Hirnforsch Expérimentation Cérébrale. 2003;153: 591–604. 10.1007/s00221-003-1616-0 14504861

[30] Maeder PP, Meuli RA, Adriani M, Bellmann A, Fornari E, Thiran JP, et al Distinct pathways involved in sound recognition and localization: a human fMRI study. NeuroImage. 2001;14: 802–816. 10.1006/nimg.2001.0888 11554799

[31] Clarke S, Bellmann Thiran A, Maeder P, Adriani M, Vernet O, Regli L, et al What and where in human audition: selective deficits following focal hemispheric lesions. Exp Brain Res Exp Hirnforsch Expérimentation Cérébrale. 2002;147: 8–15. 10.1007/s00221-002-1203-9 12373363

[32] Griffiths TD, Warren JD. What is an auditory object? Nat Rev Neurosci. 2004;5: 887–892. 10.1038/nrn1538 15496866

[33] Lewis JW, Talkington WJ, Walker NA, Spirou GA, Jajosky A, Frum C, et al Human Cortical Organization for Processing Vocalizations Indicates Representation of Harmonic Structure as a Signal Attribute. J Neurosci. 2009;29: 2283–2296. 10.1523/JNEUROSCI.4145-08.2009 19228981 PMC2774090

[34] Murray MM, Camen C, Gonzalez Andino SL, Bovet P, Clarke S. Rapid brain discrimination of sounds of objects. J Neurosci Off J Soc Neurosci. 2006;26: 1293–1302. 10.1523/JNEUROSCI.4511-05.2006 PMC6674563 16436617

[35] Warren JD, Zielinski BA, Green GGR, Rauschecker JP, Griffiths TD. Perception of sound-source motion by the human brain. Neuron. 2002;34: 139–148. 11931748 10.1016/s0896-6273(02)00637-2

[36] Altmann CF, CG de O Júnior, Heinemann L, Kaiser J. Processing of spectral and amplitude envelope of animal vocalizations in the human auditory cortex. Neuropsychologia. 2010;48: 2824–2832. 10.1016/j.neuropsychologia.2010.05.024 20493891

[37] Leaver AM, Rauschecker JP. Cortical Representation of Natural Complex Sounds: Effects of Acoustic Features and Auditory Object Category. J Neurosci. 2010;30: 7604–7612. 10.1523/JNEUROSCI.0296-10.2010 20519535 PMC2930617

[38] Schadwinkel S, Gutschalk A. Activity associated with stream segregation in human auditory cortex is similar for spatial and pitch cues. Cereb Cortex N Y N 1991. 2010;20: 2863–2873. 10.1093/cercor/bhq037 20237241

[39] Grill-Spector K. Selectivity of Adaptation in Single Units: Implications for fMRI Experiments. Neuron. 2006;49: 170–171. 10.1016/j.neuron.2006.01.004 16423690

[40] Grill-Spector K, Henson R, Martin A. Repetition and the brain: neural models of stimulus-specific effects. Trends Cogn Sci. 2006;10: 14–23. 10.1016/j.tics.2005.11.006 16321563

[41] Grill-Spector K, Kushnir T, Edelman S, Avidan G, Itzchak Y, Malach R. Differential processing of objects under various viewing conditions in the human lateral occipital complex. Neuron. 1999;24: 187–203. 10677037 10.1016/s0896-6273(00)80832-6

[42] Henson RN, Eckstein D, Waszak F, Frings C, Horner AJ. Stimulus–response bindings in priming. Trends Cogn Sci. 2014;18: 376–384. 10.1016/j.tics.2014.03.004 24768034 PMC4074350

[43] Gotts SJ, Chow CC, Martin A. Repetition priming and repetition suppression: A case for enhanced efficiency through neural synchronization. Cogn Neurosci. 2012;3: 227–237. 10.1080/17588928.2012.670617 23144664 PMC3491809

[44] Ahveninen J, Jääskeläinen IP, Raij T, Bonmassar G, Devore S, Hämäläinen M, et al Task-modulated “what” and “where” pathways in human auditory cortex. Proc Natl Acad Sci. 2006;103: 14608–14613. 10.1073/pnas.0510480103 16983092 PMC1600007

[45] Altmann CF, Bledowski C, Wibral M, Kaiser J. Processing of location and pattern changes of natural sounds in the human auditory cortex. NeuroImage. 2007;35: 1192–1200. 10.1016/j.neuroimage.2007.01.007 17320413

[46] Bourquin NM-P, Murray MM, Clarke S. Location-independent and location-linked representations of sound objects. NeuroImage. 2013;73: 40–49. 10.1016/j.neuroimage.2013.01.026 23357069

[47] Bourquin NM-P, Spierer L, Murray MM, Clarke S. Neural plasticity associated with recently versus often heard objects. NeuroImage. 2012;62: 1800–1806. 10.1016/j.neuroimage.2012.04.055 22561412

[48] De Lucia M, Cocchi L, Martuzzi R, Meuli RA, Clarke S, Murray MM. Perceptual and semantic contributions to repetition priming of environmental sounds. Cereb Cortex N Y N 1991. 2010;20: 1676–1684. 10.1093/cercor/bhp230 19906809

[49] Murray MM, Camen C, Spierer L, Clarke S. Plasticity in representations of environmental sounds revealed by electrical neuroimaging. NeuroImage. 2008;39: 847–856. 10.1016/j.neuroimage.2007.09.002 17950001

[50] Altmann CF, Nakata H, Noguchi Y, Inui K, Hoshiyama M, Kaneoke Y, et al Temporal Dynamics of Adaptation to Natural Sounds in the Human Auditory Cortex. Cereb Cortex. 2008;18: 1350–1360. 10.1093/cercor/bhm166 17893422

[51] Altmann CF, Doehrmann O, Kaiser J. Selectivity for Animal Vocalizations in the Human Auditory Cortex. Cereb Cortex. 2007;17: 2601–2608. 10.1093/cercor/bhl167 17255111

[52] Andics A, Gál V, Vicsi K, Rudas G, Vidnyánszky Z. FMRI repetition suppression for voices is modulated by stimulus expectations. NeuroImage. 2013;69: 277–283. 10.1016/j.neuroimage.2012.12.033 23268783

[53] Andics A, McQueen JM, Petersson KM, Gál V, Rudas G, Vidnyánszky Z. Neural mechanisms for voice recognition. NeuroImage. 2010;52: 1528–1540. 10.1016/j.neuroimage.2010.05.048 20553895

[54] Bergerbest D, Ghahremani DG, Gabrieli JDE. Neural Correlates of Auditory Repetition Priming: Reduced fMRI Activation in the Auditory Cortex. J Cogn Neurosci. 2004;16: 966–977. 10.1162/0898929041502760 15298784

[55] Doehrmann O, Naumer MJ, Volz S, Kaiser J, Altmann CF. Probing category selectivity for environmental sounds in the human auditory brain. Neuropsychologia. 2008;46: 2776–2786. 10.1016/j.neuropsychologia.2008.05.011 18597794

[56] Belin P, Zatorre RJ, Lafaille P, Ahad P, Pike B. Voice-selective areas in human auditory cortex. Nature. 2000;403: 309–312. 10.1038/35002078 10659849

[57] Bidet-Caulet A, Voisin J, Bertrand O, Fonlupt P. Listening to a walking human activates the temporal biological motion area. NeuroImage. 2005;28: 132–139. 10.1016/j.neuroimage.2005.06.018 16027008

[58] De Lucia M, Tzovara A, Bernasconi F, Spierer L, Murray MM. Auditory perceptual decision-making based on semantic categorization of environmental sounds. NeuroImage. 2012;60: 1704–1715. 10.1016/j.neuroimage.2012.01.131 22330317

[59] Engel LR, Frum C, Puce A, Walker NA, Lewis JW. Different categories of living and non-living sound-sources activate distinct cortical networks. NeuroImage. 2009;47: 1778–1791. 10.1016/j.neuroimage.2009.05.041 19465134 PMC2774089

[60] Giraud AL, Lorenzi C, Ashburner J, Wable J, Johnsrude I, Frackowiak R, et al Representation of the temporal envelope of sounds in the human brain. J Neurophysiol. 2000;84: 1588–1598. 10980029 10.1152/jn.2000.84.3.1588

[61] Rauschecker JP, Tian B. Mechanisms and streams for processing of “what” and “where” in auditory cortex. Proc Natl Acad Sci U S A. 2000;97: 11800–11806. 10.1073/pnas.97.22.11800 11050212 PMC34352

[62] Rauschecker JP, Tian B, Hauser M. Processing of complex sounds in the macaque nonprimary auditory cortex. Science. 1995;268: 111–114. 7701330 10.1126/science.7701330

[63] Sharda M, Singh NC. Auditory perception of natural sound categories—An fMRI study. Neuroscience. 2012;214: 49–58. 10.1016/j.neuroscience.2012.03.053 22522473

[64] Van der Zwaag W, Gentile G, Gruetter R, Spierer L, Clarke S. Where sound position influences sound object representations: a 7-T fMRI study. NeuroImage. 2011;54: 1803–1811. 10.1016/j.neuroimage.2010.10.032 20965262

[65] Woods DL, Herron TJ, Cate AD, Kang X, Yund EW. Phonological processing in human auditory cortical fields. Front Hum Neurosci. 2011;5: 42 10.3389/fnhum.2011.00042 21541252 PMC3082852

[66] Zatorre RJ, Belin P. Spectral and Temporal Processing in Human Auditory Cortex. Cereb Cortex. 2001;11: 946–953. 10.1093/cercor/11.10.946 11549617

[67] Latinus M, Taylor MJ. Discriminating male and female voices: differentiating pitch and gender. Brain Topogr. 2012;25: 194–204. 10.1007/s10548-011-0207-9 22080221

[68] Staeren N, Renvall H, De Martino F, Goebel R, Formisano E. Sound Categories Are Represented as Distributed Patterns in the Human Auditory Cortex. Curr Biol. 2009;19: 498–502. 10.1016/j.cub.2009.01.066 19268594

[69] Van der Zwaag W, Francis S, Head K, Peters A, Gowland P, Morris P, et al fMRI at 1.5, 3 and 7 T: Characterising BOLD signal changes. NeuroImage. 2009;47: 1425–1434. 10.1016/j.neuroimage.2009.05.015 19446641

[70] Van der Zwaag W, Marques JP, Lei H, Just N, Kober T, Gruetter R. Minimization of Nyquist ghosting for echo-planar imaging at ultra-high fields based on a “negative readout gradient” strategy. J Magn Reson Imaging JMRI. 2009;30: 1171–1178. 10.1002/jmri.21951 19856451

[71] De Martino F, Zimmermann J, Muckli L, Ugurbil K, Yacoub E, Goebel R. Cortical Depth Dependent Functional Responses in Humans at 7T: Improved Specificity with 3D GRASE. PLoS ONE. 2013;8: e60514 10.1371/journal.pone.0060514 23533682 PMC3606277

[72] Yacoub E, Shmuel A, Logothetis N, Ugurbil K. Robust detection of ocular dominance columns in humans using Hahn Spin Echo BOLD functional MRI at 7 Tesla. NeuroImage. 2007;37: 1161–1177. 10.1016/j.neuroimage.2007.05.020 17702606 PMC2040323

[73] De Martino F, Moerel M, Xu J, van de Moortele P-F, Ugurbil K, Goebel R, et al High-Resolution Mapping of Myeloarchitecture In Vivo: Localization of Auditory Areas in the Human Brain. Cereb Cortex N Y N 1991. 2014; 10.1093/cercor/bhu150 PMC4585494 24994817

[74] Dick F, Tierney AT, Lutti A, Josephs O, Sereno MI, Weiskopf N. In Vivo Functional and Myeloarchitectonic Mapping of Human Primary Auditory Areas. J Neurosci. 2012;32: 16095–16105. 10.1523/JNEUROSCI.1712-12.2012 23152594 PMC3531973

[75] Da Costa S, van der Zwaag W, Miller LM, Clarke S, Saenz M. Tuning In to Sound: Frequency-Selective Attentional Filter in Human Primary Auditory Cortex. J Neurosci. 2013;33: 1858–1863. 10.1523/JNEUROSCI.4405-12.2013 23365225 PMC4340971

[76] Engel SA. The development and use of phase-encoded functional MRI designs. NeuroImage. 2011; 10.1016/j.neuroimage.2011.09.059 21985909

[77] Da Costa S, Saenz M, Clarke S, Zwaag W van der. Tonotopic Gradients in Human Primary Auditory Cortex: Concurring Evidence From High-Resolution 7 T and 3 T fMRI. Brain Topogr. 2014; 1–4. 10.1007/s10548-014-0388-0 25098273

[78] Langers DRM, Dijk P van. Mapping the Tonotopic Organization in Human Auditory Cortex with Minimally Salient Acoustic Stimulation. Cereb Cortex. 2012;22: 2024–2038. 10.1093/cercor/bhr282 21980020 PMC3412441

[79] Woods DL, Alain C. Functional imaging of human auditory cortex. Curr Opin Otolaryngol Head Neck Surg. 2009;17: 407–411. 10.1097/MOO.0b013e3283303330 19633556

[80] Knebel J-F, Toepel U, Hudry J, le Coutre J, Murray MM. Generating controlled image sets in cognitive neuroscience research. Brain Topogr. 2008;20: 284–289. 10.1007/s10548-008-0046-5 18338244

[81] Salomon R, Darulova J, Narsude M, van der Zwaag W. Comparison of an 8-channel and a 32-channel coil for high-resolution FMRI at 7 T. Brain Topogr. 2014;27: 209–212. 10.1007/s10548-013-0298-6 23749257

[82] Marques JP, Kober T, Krueger G, van der Zwaag W, Van de Moortele P-F, Gruetter R. MP2RAGE, a self bias-field corrected sequence for improved segmentation and T1-mapping at high field. NeuroImage. 2010;49: 1271–1281. 10.1016/j.neuroimage.2009.10.002 19819338

[83] Goebel R, Esposito F, Formisano E. Analysis of functional image analysis contest (FIAC) data with brainvoyager QX: From single-subject to cortically aligned group general linear model analysis and self-organizing group independent component analysis. Hum Brain Mapp. 2006;27: 392–401. 10.1002/hbm.20249 16596654 PMC6871277

[84] Larsson J, Smith AT. fMRI Repetition Suppression: Neuronal Adaptation or Stimulus Expectation? Cereb Cortex. 2012;22: 567–576. 10.1093/cercor/bhr119 21690262 PMC3278317

[85] Lewis JW, Talkington WJ, Walker NA, Spirou GA, Jajosky A, Frum C, et al Human Cortical Organization for Processing Vocalizations Indicates Representation of Harmonic Structure as a Signal Attribute. J Neurosci. 2009;29: 2283–2296. 10.1523/JNEUROSCI.4145-08.2009 19228981 PMC2774090

[86] Hackett TA, Barkat TR, O’Brien BMJ, Hensch TK, Polley DB. Linking topography to tonotopy in the mouse auditory thalamocortical circuit. J Neurosci Off J Soc Neurosci. 2011;31: 2983–2995. 10.1523/JNEUROSCI.5333-10.2011 21414920 PMC3073837

[87] Lewis JW, Brefczynski JA, Phinney RE, Janik JJ, DeYoe EA. Distinct cortical pathways for processing tool versus animal sounds. J Neurosci Off J Soc Neurosci. 2005;25: 5148–5158. 10.1523/JNEUROSCI.0419-05.2005 PMC6724809 15917455

[88] Specht K, Reul J. Functional segregation of the temporal lobes into highly differentiated subsystems for auditory perception: an auditory rapid event-related fMRI-task. NeuroImage. 2003;20: 1944–1954. 14683700 10.1016/j.neuroimage.2003.07.034

[89] Lewis JW, Talkington WJ, Tallaksen KC, Frum CA. Auditory object salience: human cortical processing of non-biological action sounds and their acoustic signal attributes. Front Syst Neurosci. 2012;6: 27 10.3389/fnsys.2012.00027 22582038 PMC3348722

[90] Belin P, Zatorre RJ. Adaptation to speaker’s voice in right anterior temporal lobe. Neuroreport. 2003;14: 2105–2109. 10.1097/01.wnr.0000091689.94870.85 14600506

[91] Renvall H, Staeren N, Siep N, Esposito F, Jensen O, Formisano E. Of cats and women: Temporal dynamics in the right temporoparietal cortex reflect auditory categorical processing of vocalizations. NeuroImage. 2012;62: 1877–1883. 10.1016/j.neuroimage.2012.06.010 22721629

[92] Chandrasekaran B, Chan AHD, Wong PCM. Neural processing of what and who information in speech. J Cogn Neurosci. 2011;23: 2690–2700. 10.1162/jocn.2011.21631 21268667

[93] Giordano BL, McAdams S, Zatorre RJ, Kriegeskorte N, Belin P. Abstract Encoding of Auditory Objects in Cortical Activity Patterns. Cereb Cortex. 2013;23: 2025–2037. 10.1093/cercor/bhs162 22802575

[94] Warren JD, Griffiths TD. Distinct mechanisms for processing spatial sequences and pitch sequences in the human auditory brain. J Neurosci Off J Soc Neurosci. 2003;23: 5799–5804. 10.1523/JNEUROSCI.23-13-05799.2003 PMC6741275 12843284

[95] Hart HC, Palmer AR, Hall DA. Different areas of human non‐primary auditory cortex are activated by sounds with spatial and nonspatial properties. Hum Brain Mapp. 2004;21: 178–190. 10.1002/hbm.10156 14755837 PMC6872110

[96] Hall DA, Barrett DJK, Akeroyd MA, Summerfield AQ. Cortical representations of temporal structure in sound. J Neurophysiol. 2005;94: 3181–3191. 10.1152/jn.00271.2005 16014796

[97] Barrett DJK, Hall DA. Response preferences for “what” and “where” in human non-primary auditory cortex. NeuroImage. 2006;32: 968–977. 10.1016/j.neuroimage.2006.03.050 16733092

[98] Griffiths TD, Warren JD. The planum temporale as a computational hub. Trends Neurosci. 2002;25: 348–353. 10.1016/S0166-2236(02)02191-4 12079762

[99] Schadwinkel S, Gutschalk A. Functional dissociation of transient and sustained fMRI BOLD components in human auditory cortex revealed with a streaming paradigm based on interaural time differences. Eur J Neurosci. 2010;32: 1970–1978. 10.1111/j.1460-9568.2010.07459.x 21050277

[100] Smith KR, Hsieh I-H, Saberi K, Hickok G. Auditory spatial and object processing in the human planum temporale: no evidence for selectivity. J Cogn Neurosci. 2010;22: 632–639. 10.1162/jocn.2009.21196 19301992

[101] Zündorf IC, Karnath H-O, Lewald J. The effect of brain lesions on sound localization in complex acoustic environments. Brain. 2014;137: 1410–1418. 10.1093/brain/awu044 24618271

[102] Cammoun L, Thiran JP, Griffa A, Meuli R, Hagmann P, Clarke S. Intrahemispheric cortico-cortical connections of the human auditory cortex. Brain Struct Funct. 2014; 1–17. 10.1007/s00429-014-0872-z 25173473

[103] Clarke S, Bellmann A, De Ribaupierre F, Assal G. Non-verbal auditory recognition in normal subjects and brain-damaged patients: evidence for parallel processing. Neuropsychologia. 1996;34: 587–603. 8736571 10.1016/0028-3932(95)00142-5

