# Supplementary figures and images for: Representation of Sound Objects within Early-Stage Auditory Areas: A Repetition Effect Study Using 7T fMRI
Source: PLoS One. 2015 May 4;10(5):e0124072. doi: 10.1371/journal.pone.0124072 (PMC4418571; doi:10.1371/journal.pone.0124072)

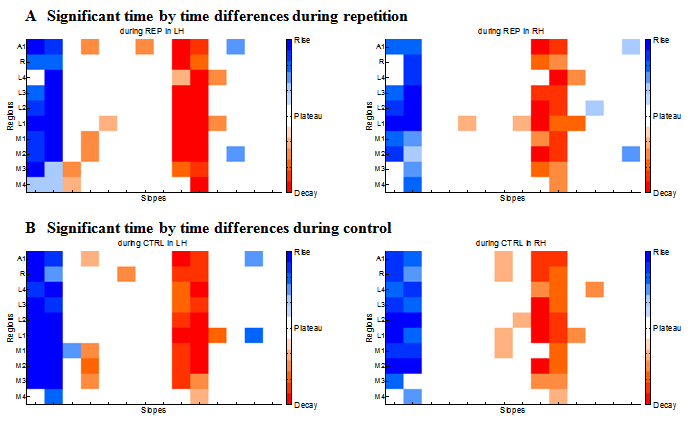

Supplement: S1 Fig — A—B. Plateau definition. Temporal derivatives of the averaged time-courses for each ROI illustrate the slope between two consecutive time–points. Paired t-tests of the derivatives against 0 pointed out three different periods: rise from 2 to 6 s, plateau from 6 to 18 s, and decay from 18 to 22 s. Shades of blue correspond to positive slopes (rise), orange-red to negative slopes (decay) and white to zero-gradient parts of the curves. (TIF) [file pone.0124072.s001.tif]

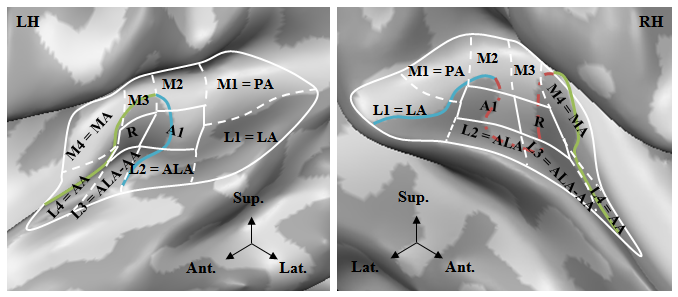

Supplement: S2 Fig — For this exemplar subject, the left hemisphere had a single HG and the right hemisphere a complete duplication. 10 ROIs were defined based on the tonotopic gradients for each hemisphere: M1, L1, M2, A1, L2, M3, R, L3, M4, and L4. Several ROIs corresponded to the auditory areas found in the architectonic studies of Rivier and Clarke (1997) and Wallace et al. (2002). Blue line: anterior Heschl’s gyrus border; green line: posterior Heschl’s gyrus border; red line: intermediate sulcus. (TIF) [file pone.0124072.s002.tif]

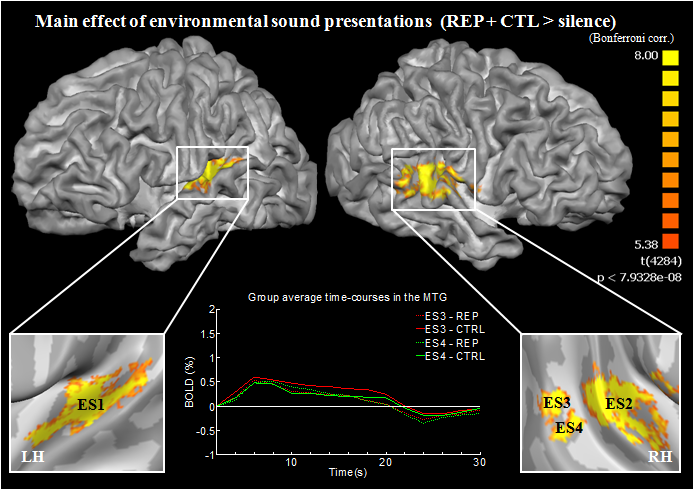

Supplement: S3 Fig — The group average fixed-effect multi-subject GLM contrast environmental sounds vs. rest (i.e. REP + CTRL > rest) revealed activation outside the auditory cortex (S2 Fig, ES1, ES2) on both sides in the posterior superior temporal gyrus (STG), posterior middle temporal gyrus (MTG; S2 Fig and S1 Table; ES3 area: 362.59 mm2 and ES4 area: 308.11 mm2; p<0.05, Bonferroni correction). As for individual ROIs, the group ROIs were labelled with their region name and projected into the reference brain 1 x 1 x 1 mm interpolated volumetric space. Individual time courses of these regions were subsequently analyzed in the repetition suppression experiment. Time-courses of ES3 and ES4 are plotted for each condition in the graph. ES3 and ES4 ROIs showed both the same tendency, with higher BOLD response during control blocks, but none showed significant differences. It is to be noted that the group average fixed-effect multi-subject GLM constrast REP vs CTRL did not show any significant difference (p>0.05, Bonferroni correction). Upper panel: significant activation clusters (p<0.05, Bonferroni corrected). Lower panels: enlargement of the activated regions on a partially inflated brain. Environmental sounds activated two large clusters within the STG (ES1 and ES2), but also two smaller clusters in the right posterior MTG (ES3 and ES4). Mean time courses for these latter clusters are plotted in red and green in the graph between the two enlargements. Time frame by time frame analysis revealed no significant differences between the two conditions surviving the inclusion criteria. (TIF) [file pone.0124072.s003.tif]

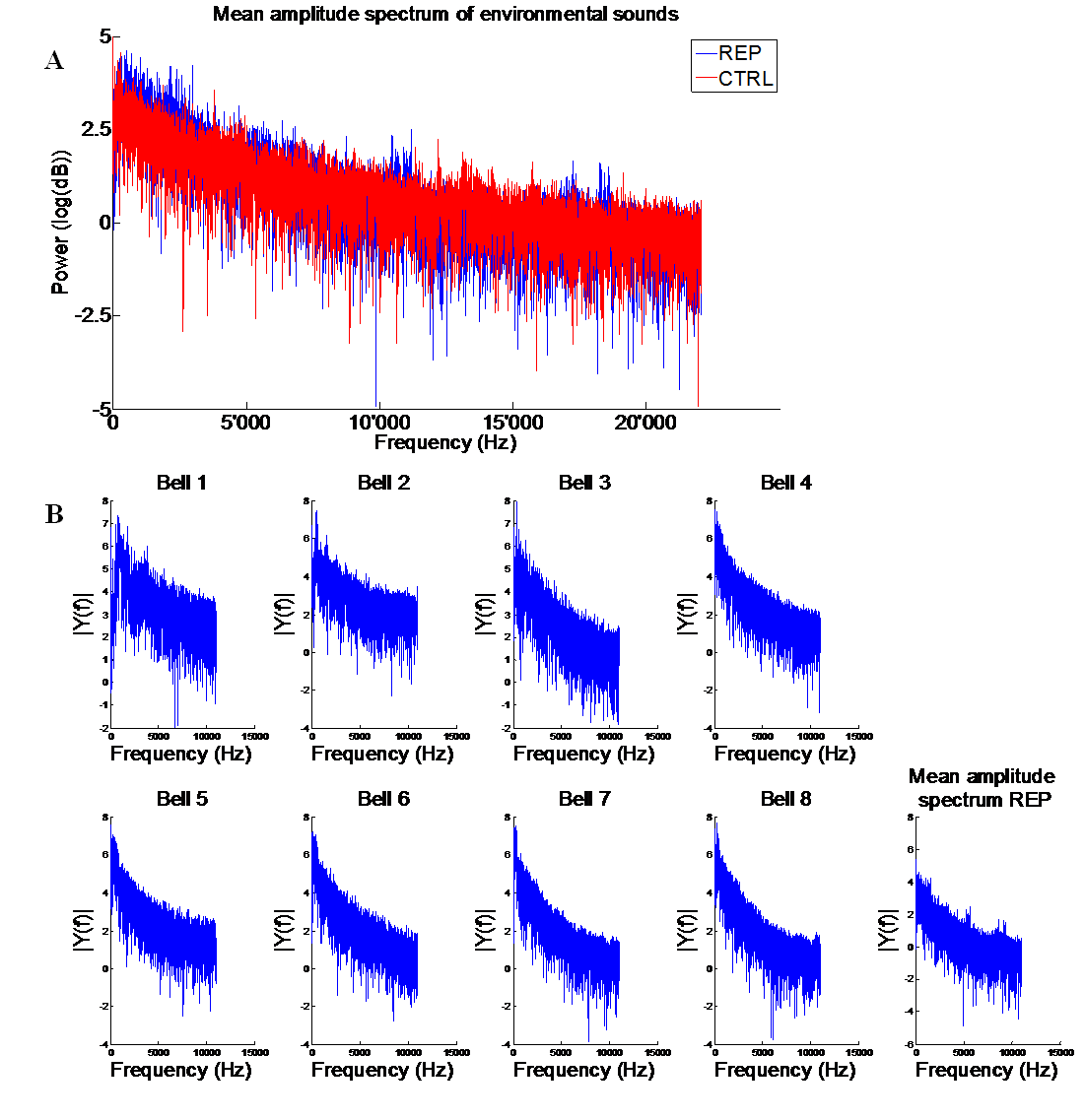

Supplement: S4 Fig — A. Mean amplitude spectrum of the environmental sounds used in the paradigm. Each amplitude spectrum of the sounds of the two conditions has been decomposed using a fast Fourier transform function and plotted across the frequency range from 0 to 25000 Hz. Blue line: mean amplitude spectrum for the repetition group sounds; red line: mean amplitude spectrum for the control group sounds. Unpaired t-tests between the amplitude spectra of both conditions for each frequency revealed that 110 non-consecutive frequencies were significantly different between conditions, which corresponded to 1% after Bonferroni correction (110/11025 = 0.01). B. Amplitude spectrum of each sound in a REP block where eight different bell sounds were presented. Frequency distributions within a block are different in each exemplar compared to the mean amplitude spectrum of REP condition (bottom right graph). (TIF) [file pone.0124072.s004.tif]
