# Supplementary material for: Representation of Sound Objects within Early-Stage Auditory Areas: A Repetition Effect Study Using 7T fMRI
Source: PLoS One. 2015 May 4;10(5):e0124072. doi: 10.1371/journal.pone.0124072 (PMC4418571; doi:10.1371/journal.pone.0124072)
Supplement: S1 Table — Only sounds correctly recognized during the sound recognition pilot by five subjects were used in the fMRI experiment. All sounds of the REP group (8 sound objects) were used in the fMRI runs, whereas only one exemplar of each sound object was randomly selected in the CTRL group (64 sounds objects). The REP groups was the same in all subjects, whereas the CTRL group varied in all subjects. human voc.: human vocalizations; human non-voc.: human non-vocalizations; env. sound: environmental sound. (DOCX) [file pone.0124072.s005.docx]

**Table S1. Environmental sounds used in the repetition suppression paradigm.**

| **REP BLOCKS** |  |  |  |  |  |
| --- | --- | --- | --- | --- | --- |
| **File name** | **Categorie** | **File name** | **Categorie** | **File name** | **Categorie** |
| baby1 | human voc. | bell9 | instrument | dog5 | animal voc. |
| baby2 | human voc. | bell10 | instrument | dog8 | animal voc. |
| baby3 | human voc. | bubbles1 | env. sound | dog9 | animal voc. |
| baby4 | human voc. | bubbles2 | env. sound | dog10 | animal voc. |
| baby5 | human voc. | bubbles4 | env. sound | duck1 | animal voc. |
| baby6 | human voc. | bubbles5 | env. sound | duck2 | animal voc. |
| baby8 | human voc. | bubbles6 | env. sound | duck3 | animal voc. |
| baby9 | human voc. | bubbles7 | env. sound | duck4 | animal voc. |
| bagpipe2 | instrument | bubbles8 | env. sound | duck5 | animal voc. |
| bagpipe3 | instrument | bubbles10 | env. sound | duck6 | animal voc. |
| bagpipe4 | instrument | cat1 | animal voc. | duck7 | animal voc. |
| bagpipe6 | instrument | cat2 | animal voc. | duck8 | animal voc. |
| bagpipe7 | instrument | cat3 | animal voc. | human_shout1 | human voc. |
| bagpipe10 | instrument | cat4 | animal voc. | human_shout2 | human voc. |
| bagpipe11 | instrument | cat6 | animal voc. | human_shout3 | human voc. |
| bagpipe12 | instrument | cat8 | animal voc. | human_shout4 | human voc. |
| bell1 | instrument | cat11 | animal voc. | human_shout5 | human voc. |
| bell2 | instrument | cat12 | animal voc. | human_shout6 | human voc. |
| bell3 | instrument | dog1 | animal voc. | human_shout7 | human voc. |
| bell4 | instrument | dog2 | animal voc. | human_shout8 | human voc. |
| bell5 | instrument | dog3 | animal voc. |  |  |
| bell6 | instrument | dog4 | animal voc. |  |  |
|  |  |  |  |  |  |
| **CTRL BLOCKS** |  |  |  |  |  |
| **File name** | **Categorie** | **File name** | **Categorie** | **File name** | **Categorie** |
| accordion1 | instrument | bike_bell4 | tool | crow2 | animal voc. |
| accordion2 | instrument | car1 | vehicle | crow3 | animal voc. |
| accordion3 | instrument | cash_machine1 | tool | crow6 | animal voc. |
| accordion4 | instrument | chicken1 | animal voc. | crunching1 | human non-voc. |
| accordion5 | instrument | chicken2 | animal voc. | crunching2 | human non-voc. |
| accordion6 | instrument | chicken3 | animal voc. | crunching3 | human non-voc. |
| accordion7 | instrument | chicken4 | animal voc. | crunching4 | human non-voc. |
| alarm1 | tool | chicken5 | animal voc. | crunching5 | human non-voc. |
| alarm2 | tool | coughing1 | human non-voc. | crunching6 | human non-voc. |
| applauses1 | human non-voc. | coughing2 | human non-voc. | crunching8 | human non-voc. |
| applauses2 | human non-voc. | coughing4 | human non-voc. | cuckoo_clock1 | env. sound |
| applauses3 | human non-voc. | coughing5 | human non-voc. | cuckoo1 | animal voc. |
| applauses4 | human non-voc. | cow_mooing1 | animal voc. | cymballe1 | instrument |
| applauses5 | human non-voc. | cow_mooing2 | animal voc. | cymballe2 | instrument |
| applauses6 | human non-voc. | creaking_door1 | env. sound | donkey1 | animal voc. |
| bee1 | animal voc. | creaking_door2 | env. sound | donkey2 | animal voc. |
| bee3 | animal voc. | creaking_door3 | env. sound | door_knocking1 | env. sound |
| bee4 | animal voc. | creasing_paper4 | env. sound | door_knocking2 | env. sound |
| bike_bell1 | tool | cricket2 | animal voc. | doorbell1 | env. sound |
| bike_bell2 | tool | cricket3 | animal voc. | doorbell2 | env. sound |
| bike_bell3 | tool | crow1 | animal voc. | double_bass1 | instrument |
| **File name** | **Categorie** | **File name** | **Categorie** | **File name** | **Categorie** |
| double_bass2 | instrument | hammer4 | tool | plane2 | env. sound |
| double_bass3 | instrument | harmonica1 | instrument | plane3 | env. sound |
| double_bass4 | instrument | harmonica2 | instrument | plane4 | env. sound |
| double_bass5 | instrument | harmonica3 | instrument | river1 | env. sound |
| double_bass6 | instrument | harmonica4 | instrument | river2 | env. sound |
| double_bass7 | instrument | harmonica5 | instrument | river3 | env. sound |
| drums1 | instrument | helicopter1 | env. sound | river6 | env. sound |
| drums2 | instrument | helicopter2 | env. sound | river7 | env. sound |
| drums3 | instrument | helicopter3 | env. sound | river8 | env. sound |
| drums4 | instrument | helicopter4 | env. sound | river9 | env. sound |
| drums5 | instrument | horn1 | tool | rooster1 | animal voc. |
| elephant_noise1 | animal voc. | horn2 | tool | rooster2 | animal voc. |
| elephant_noise3 | animal voc. | horn3 | tool | rooster3 | animal voc. |
| explosion1 | env. sound | horn4 | tool | rooster4 | animal voc. |
| explosion2 | env. sound | horn5 | tool | saw1 | tool |
| fart1 | human non-voc. | horse1 | animal voc. | saw2 | tool |
| fire1 | env. sound | horse2 | animal voc. | saw7 | tool |
| fire2 | env. sound | horse3 | animal voc. | saw9 | tool |
| fire3 | env. sound | indian_song1 | human voc. | saw10 | tool |
| fire4 | env. sound | indian_song2 | human voc. | saw11 | tool |
| footsteps11 | human non-voc. | indian_song3 | human voc. | saxophone2 | instrument |
| frog1 | animal voc. | lion5 | animal voc. | saxophone3 | instrument |
| frog2 | animal voc. | lion6 | animal voc. | saxophone4 | instrument |
| gargling1 | human non-voc. | man_voc1 | human voc. | scissors1 | tool |
| gargling2 | human non-voc. | man_voc2 | human voc. | scissors2 | tool |
| gargling3 | human non-voc. | man_voc3 | human voc. | scissors3 | tool |
| gargling4 | human non-voc. | monkey1 | animal voc. | scrubbing_brush7 | tool |
| glass_breaking3 | env. sound | monkey2 | animal voc. | seal1 | animal voc. |
| glass_filling1 | env. sound | monney_drop1 | env. sound | seal3 | animal voc. |
| glass_filling2 | env. sound | monney_drop2 | env. sound | seal4 | animal voc. |
| glass_filling3 | env. sound | monney_drop3 | env. sound | seal5 | animal voc. |
| glass_filling4 | env. sound | monney_drop4 | env. sound | seal6 | animal voc. |
| glass_filling5 | env. sound | monney_drop5 | env. sound | seal7 | animal voc. |
| glass_filling6 | env. sound | monney_drop6 | env. sound | sharpen_knife1 | tool |
| glasses_clinking1 | env. sound | monney_drop7 | env. sound | sharpen_knife2 | tool |
| glasses_clinking3 | env. sound | nose_blowing1 | human non-voc. | sharpen_knife3 | tool |
| guitar1 | instrument | organ1 | instrument | sharpen_knife5 | tool |
| guitar2 | instrument | organ2 | instrument | sheep1 | animal voc. |
| guitar6 | instrument | organ3 | instrument | sheep2 | animal voc. |
| guitar9 | instrument | organ4 | instrument | sipping1 | human non-voc. |
| guitar10 | instrument | owl1 | animal voc. | sneezing1 | human non-voc. |
| gun_firing1 | env. sound | owl3 | animal voc. | sneezing2 | human non-voc. |
| gun_firing2 | env. sound | owl4 | animal voc. | sneezing3 | human non-voc. |
| gun_firing3 | env. sound | page_turning1 | env. sound | snoring3 | human non-voc. |
| gun_firing4 | env. sound | page_turning2 | env. sound | spray3 | env. sound |
| gun_firing5 | env. sound | page_turning3 | env. sound | sword1 | tool |
| gun_firing6 | env. sound | page_turning4 | env. sound | tarzan1 | human voc. |
| gun_firing7 | env. sound | pig1 | animal voc. | tarzan2 | human voc. |
| **File name** | **Categorie** | **File name** | **Categorie** | **File name** | **Categorie** |
| tarzan3 | human voc. | typing1 | tool | wind5 | env. sound |
| telephone_ring1 | tool | typing2 | tool | wolf1 | animal voc. |
| telephone_ring2 | tool | typing3 | tool | woman_voc1 | human voc. |
| telephone_ring3 | tool | waves_sound4 | env. sound | woman_voc3 | human voc. |
| telephone_ring4 | tool | waves_sound5 | env. sound | woman_voc4 | human voc. |
| telephone_ring5 | tool | waves_sound6 | env. sound | woman_voc5 | human voc. |
| tenis_ball1 | env. sound | waves_sound7 | env. sound | woman_voc6 | human voc. |
| tenis_ball2 | env. sound | waves_sound8 | env. sound | woman_voc7 | human voc. |
| tenis_ball3 | env. sound | whistle1 | human non-voc. | woman_voc9 | human voc. |
| tenis_ball4 | env. sound | whistle2 | human non-voc. | yawn1 | human non-voc. |
| tenis_ball5 | env. sound | whistle3 | human non-voc. | yawn3 | human non-voc. |
| tenis_ball6 | env. sound | whistle4 | human non-voc. | yawn4 | human non-voc. |
| tic-tac_clock1 | env. sound | whistle5 | human non-voc. | zipper1 | env. sound |
| tic-tac_clock2 | env. sound | whistle6 | human non-voc. | zipper2 | env. sound |
| trombone2 | instrument | wind1 | env. sound | zipper3 | env. sound |
| trombone3 | instrument | wind2 | env. sound |  |  |
| trombone4 | instrument | wind3 | env. sound |  |  |
| trombone5 | instrument | wind4 | env. sound |  |  |

Only sounds correctly recognized during the sound recognition pilot by five subjects were used in the fMRI experiment. All sounds of the REP group (8 sound objects) were used in the fMRI runs, whereas only one exemplar of each sound object was randomly selected in the CTRL group (64 sounds objects). The REP groups was the same in all subjects, whereas the CTRL group varied in all subjects. human voc.: human vocalizations; human non-voc.: human non-vocalizations; env. sound: environmental sound.
