# Supplementary material for: Representation of Sound Objects within Early-Stage Auditory Areas: A Repetition Effect Study Using 7T fMRI
Source: PLoS One. 2015 May 4;10(5):e0124072. doi: 10.1371/journal.pone.0124072 (PMC4418571; doi:10.1371/journal.pone.0124072)
Supplement: S2 Table — Centre coordinates of the activation clusters shown in S3 Fig, t scores, and p values. Only regions that remained significant at p<0.05 after application of the Bonferroni correction were considered. (DOCX) [file pone.0124072.s006.docx]

Table S2. Main effect of the environmental sound presentation (REP + CTRL > silence).

|  | **Region** | **Talairach coordinates**  [X Y Z] ± std | **t score** | **p value** |
| --- | --- | --- | --- | --- |
| **ES1** | left STG | [ -52 -23 7] | 17.12 | p < 0.00 ** |
| **ES2** | right STG | [ 60 -24 13] | 15.23 | p < 0.00 ** |
| **ES3** | right posterior MTG | [ 56 -35 15] | 12.49 | p < 0.00 ** |
| **ES4** | right posterior MTG | [ 61 -32 4] | 9.31 | p < 0.00 ** |

Centre coordinates of the activation clusters shown in Figure S3, t scores, and p values. Only regions that remained significant at p < 0.05 after application of the Bonferroni correction were considered.
