# Supplementary material for: Representation of Sound Objects within Early-Stage Auditory Areas: A Repetition Effect Study Using 7T fMRI
Source: PLoS One. 2015 May 4;10(5):e0124072. doi: 10.1371/journal.pone.0124072 (PMC4418571; doi:10.1371/journal.pone.0124072)
Supplement: S3 Table — Paired t-tests between REP vs CTRL maxima ([max]) revealed significant differences in right A1(1), right M1(2), right M2(3), right M4(4), left A1(5), and left M3(6) (p<0.05, uncorrected). Paired t-tests between REP vs CTRL minima ([min]) revealed significant differences in right R(7), left A1(8) and left M3(9) (p<0.05, uncorrected). Paired t-tests between RH vs LH maxima during REP and RH vs LH minima during CTRL revealed significant differences in M2(10) and L2(11), respectively (p<0.05, uncorrected). No significant differences were found for the amplitudes. (DOCX) [file pone.0124072.s007.docx]

Table S3. Maxima, minima and amplitudes of the BOLD response during REP and CTRL in both hemispheres. Paired t-tests between REP *vs* CTRL maxima (*[max]*) revealed significant differences in right A1^(1)^, right M1^(2)^, right M2^(3)^, right M4^(4)^, left A1^(5)^, and left M3^(6)^ ( p < 0.05, uncorrected). Paired t-tests between REP *vs* CTRL minima (*[min]*) revealed significant differences in right R^(7)^, left A1^(8)^ and left M3^(9)^ (p < 0.05, uncorrected). Paired t-tests between RH *vs* LH maxima during REP and RH *vs* LH minima during CTRL revealed significant differences in M2^(10)^ and L2^(11)^, respectively (p < 0.05, uncorrected). No significant differences were found for the amplitudes.

| ***Labels*** | ***During REP*** | | | | ***During CTRL*** | | | |
| --- | --- | --- | --- | --- | --- | --- | --- | --- |
|  | *[max ] ± std* | *[min] ± std* | *[amp] ± std* | *[max] ± std* | | *[min] ± std* | *[amp] ± std* |  |
| ***Right hemisphere*** | | | | | | | | |
| right A1 | **1.27 ± 0.27^(1)^** | -0.85 ± 0.16 | 2.12 ± 0.40 | **1.60 ± 0.39^(1)^** | | -0.59 ± 0.13 | 2.19 ± 0.45 |  |
| right R | 1.07 ± 0.24 | **-0.85 ± 0.21^(7)^** | 1.92 ± 0.42 | 1.37 ± 0.33 | | **-0.52 ± 0.17^(7)^** | 1.90 ± 0.44 |  |
| right L1 | 1.41 ± 0.26 | -0.53 ± 0.15 | 1.94 ± 0.39 | 1.61 ± 0.32 | | -0.38 ± 0.10 | 1.99 ± 0.35 |  |
| right L2 | 1.22 ± 0.21 | -0.51 ± 0.16 | 1.73 ± 0.32 | 1.21 ± 0.25 | | **-0.51 ± 0.15^(11)^** | 1.72 ± 0.31 |  |
| right L3 | 1.03 ± 0.20 | -0.46 ± 0.15 | 1.49 ± 0.30 | 1.08 ± 0.24 | | -0.38 ± 0.13 | 1.46 ± 0.25 |  |
| right L4 | 0.77 ± 0.19 | -0.31 ± 0.02 | 1.09 ± 0.19 | 0.81 ± 0.16 | | -0.20 ± 0.05 | 1.02 ± 0.17 |  |
| right M1 | **0.86 ± 0.15^(2)^** | -0.40 ± 0.10 | 1.26 ± 0.21 | **1.12 ± 0.19^(2)^** | | -0.35 ± 0.11 | 1.47 ± 0.25 |  |
| right M2 | **0.73 ± 0.09^(3&10)^** | -0.58 ± 0.15 | 1.31 ± 0.21 | **0.99 ± 0.16^(3)^** | | -0.46 ± 0.13 | 1.45 ± 0.23 |  |
| right M3 | 0.74 ± 0.13 | -0.68 ± 0.17 | 1.41 ± 0.24 | 0.98 ± 0.20 | | -0.58 ± 0.17 | 1.56 ± 0.31 |  |
| right M4 | **0.50 ± 0.10^(4)^** | -0.60 ± 0.15 | 1.10 ± 0.17 | **0.69 ± 0.16^(4)^** | | -0.37 ± 0.10 | 1.06 ± 0.13 |  |
| ***Left hemisphere*** | | | | | | | | |
| left A1 | **1.29 ± 0.24^(5)^** | **-0.81 ± 0.21^(8)^** | 2.10 ± 0.39 | **1.68 ± 0.29^(5)^** | | **-0.40 ± 0.11^(8)^** | 2.08 ± 0.35 |  |
| left R | 0.98 ± 0.19 | -0.61 ± 0.16 | 1.59 ± 0.27 | 1.14 ± 0.19 | | -0.44 ± 0.07 | 1.57 ± 0.22 |  |
| left L1 | 1.40 ± 0.19 | -0.52 ± 0.14 | 1.92 ± 0.24 | 1.51 ± 0.16 | | -0.31 ± 0.06 | 1.82 ± 0.18 |  |
| left L2 | 1.20 ± 0.20 | -0.40 ± 0.11 | 1.59 ± 0.20 | 1.20 ± 0.17 | | **-0.34 ± 0.10^(11)^** | 1.54 ± 0.20 |  |
| left L3 | 0.82 ± 0.16 | -0.33 ± 0.08 | 1.16 ± 0.19 | 0.79 ± 0.14 | | -0.25 ± 0.07 | 1.05 ± 0.16 |  |
| left L4 | 0.83 ± 0.11 | -0.38 ± 0.08 | 1.21 ± 0.12 | 1.03 ± 0.12 | | -0.15 ± 0.07 | 1.18 ± 0.14 |  |
| left M1 | 1.17 ± 0.16 | -0.58 ± 0.12 | 1.75 ± 0.23 | 1.29 ± 0.17 | | -0.30 ± 0.10 | 1.60 ± 0.17 |  |
| left M2 | **1.03 ± 0.14^(10)^** | -0.68 ± 0.12 | 1.70 ± 0.17 | 1.16 ± 0.15 | | -0.47 ± 0.11 | 1.63 ± 0.13 |  |
| left M3 | **0.66 ± 0.09^(6)^** | **-0.68 ± 0.14^(9)^** | 1.34 ± 0.21 | **0.88 ± 0.13^(6)^** | | **-0.40 ± 0.14^(9)^** | 1.28 ± 0.22 |  |
| left M4 | 0.51 ± 0.09 | -0.55 ± 0.14 | 1.06 ± 0.14 | 0.67 ± 0.13 | | -0.34 ± 0.09 | 1.01 ± 0.17 |  |
